# Supplementary material for: Small area estimation and childhood obesity surveillance using electronic health records
Source: PLoS One. 2021 Feb 19;16(2):e0247476. doi: 10.1371/journal.pone.0247476 (PMC7895416; doi:10.1371/journal.pone.0247476)
Supplement: Attachment — Submitted filename: Response_Plosone__01_21_2021.docx [file pone.0247476.s001.docx]

*Reviewer #1: The present manuscript investigates the use of electronic health records for obesity surveillance, particularly at a regionally small-scale level.

The introduction presents sufficient information to understand how the authors planned to overcome the lack of regional information on obesity prevalence using electronic health records and new datasets to validate HER for surveillance purposes.

Methods are thoroughly described and provide transparent information on scores and necessary steps in data management and statistical analysis. In particular, the two step procedure is an interesting approach to deal with missing data from health records and to estimate the obesity prevalence.*

Thank you very much for the constructive comments. Point-by-point responses are given below.

*My major concern is the presentation of the results without tables of values or descriptive statistics about Prevalences, differences in prevalence, or Results from the logistics regressions to missingness in BMI. The authors chose to present crude results in Fig2 and Fig1 should be placed in the method section.*

**Response**: We have provided new tables, presenting the results from generalized linear mixed model for predicting obesity at the subject level, and prevalence and differences in prevalence for separate demographic group. See Tables 2 and 3.

*First, Fig2 needs slightly more explanation to the legend itself. Second. Fig1 should include some orientation (State within the US, Dane county, Milwauke area etc.) and better highlights the differences in prevalence. This should not be explained in the Discussion, but in the method section. If needed, other statistics of the results might be presented also as a map, but due to the large number of counties (zip codes?) it is hard to verify the picture.*

**Response**: We have modified the caption of Fig 2 to “Scatter plot showing prevalence difference estimates versus zip code similarity indices between Wisconsin Health Atlas and University of Wisconsin Public Health Information Exchange.” We have added a figure that presents the differences in prevalence as a map, see Figure 3. The explanation of Fig 1 has been moved to the method section.

*As a rule of thumb, most of the numbers/results presented in the results section should be presented in a table that comprises these interesting results.*

**Response**: We have provided new tables, presenting the results from generalized linear mixed model for predicting obesity at the subject level, and prevalence and differences in prevalence for separate demographic group. See Tables 2 and 3.

*The discussion is balanced and highlights benefits and drawbacks of this approach or explains them due to differences in data sources. The authors should close the discussion with one or two general statements on the use of EHR for surveillance that will be kept in mind after reading.*

**Response**: We have added the following statements to close the discussion.

EHR-based surveillance provides new opportunities and challenges. It could promote collaborations between clinical services, public health officials and other areas, facilitate timely decision support tools through the EHRs. The sufficiency regarding the population coverage and risk factor information with EHRs remain an issue to ensure representative, population-wide data. These limitations should be investigated and addressed in the future. See the paragraph starting line 315.

Some minor comments apply.

Line 70 – 73: Please state the number subjects that is mentioned in table 1 also in the first paragraph about PHINEX
Table 1: The table might provide further insights, if descriptive statistics are also presented for age or sex strata.

**Response**: Thank you for the suggestions. We have made changes, including mentioning the number of subjects in the first paragraph, and adding sex strata to the descriptive statistics. See line 92 and Table 1.

Line 98 – 101: The categorization of Urbanicity is not clear. Please further explain the 11 Groups which are somehow a score that can be categorized. How is this score roughly derived?

**Response:** We have updated the reference link (reference 17), <https://www.esri.com/news/ArcUser/0104/tapestry.html>, and provided additional descriptions of the classifications. For these analyses, urbanicity was categorized as: “urban” for 1–4 (principal urban centers 1&2; metro cities 1&2), “suburban” for 5–8 (urban outskirts 1&2; suburban periphery 1&2), and “rural” for 9–11 (small towns, rural 1 &2). The two designations for principal urban, metro cities, urban outskirts, suburban periphery, and rural areas denote relative affluence within the group. See lines 104-110.

Line 151f.: Please add also the version of package or function that you used in R.

**Response**: We have added details on the version of R packages. For our analysis, we used lme4 (version 1.1-21), ggplot2 (version 3.2.0), maps (version 3.3.0), maptools (version 0.9-5), mapdata (version 2.3.0). See lines 160-162.

Line 157f.: I might have missed it, but please specify, if you used the similar approach for the data from the Wisconsin Health Atlas for the reader. Or was data sufficient to calculate actual prevalence? I guess not, since these also based on EHR.

**Response**: We have clarified as a limitation that we did not apply the proposed approach to the Wisconsin Health Atlas (WHA) data, since we were not able to obtain access to the individual level data from WHA, see lines 261-262. We compared our results to existing local aggregate results from Wisconsin Health Atlas dataset (reference 13. Wisconsin Health Atlas. Obesity 2015–2016 Findings. Wisconsin Health Atlas. https://www.wihealthatlas.org/obesity/findings. Accessed May 10, 2019), see line 199.

*Reviewer #2: The authors present a statistical method to estimate obesity prevalence based on EHR data from Wisconsin. The underlying aim is to make more use of existing and collected EHR data. Thus, authors compare their own results with the published Wisconsin Health Atlas estimates. Not a statistician myself, I prefer not to evaluate the methods applied. However, from a non-statistician point of view, the methods seem sound and aspects such as missingness, non-randomness have been addressed, as well as Bootstrapping to account for variability in obesity and non-random data availability. Overall, the paper reads well and is of public health interest questions and has some limitations to be addressed or better discussed.*

Thank you for taking the time to review our manuscript and for your thoughtful comments. Point-by- point responses are given below.

Major points:
Authors do not discuss the limitations of their study sufficiently, such as the fact that the data they compare do not stem from the same period, nor the origin of the data, essentially EHR in both, but PHINEX from academic centers. The reviewer suggests the authors explain the choice of time periods investigated. The comparability is limited.
Differences in populations in PHINEX and WHA are assumed to be different with respect to SES and health behavior by the authors based on the geographical distribution of respective data. What is the basis of this assumption? Are these differences sufficiently corrected for with the methodology applied?

*If I understand correctly, authors did not use WHA data themselves but results of the atlas. Differences in estimates based on time period, geographical distribution and data missingness could be investigated by performing the same methods using WHA data. Please comment.*

**Response**: Thank you for the suggestions. Unfortunately, we were not able to obtain access to the individual level data from WHA. This limit our comparison to only using existing results, and indeed is a limitation of our study, and we have noted it in the discussion section. See lines 261-262.

*How was the fitted prevalence of non-missingness estimated, did authors refer to other data on children in Wisconsin to inform the analyses on prevalence of the characteristics?*

**Response**: We have clarified that the prevalence of non-missingness was estimated using the PHINEX data. Then they are inverse weighted by the probability of being not missing. See line 149.

*The method section should include the restricted analyses in Dane county. It is repeatedly singled out in the results and discussion section. Does the Dane county correspond to one zip code? Why is Dane county so relevant, authors should explain in more detail?*

**Response**: We have conducted the restricted analyses in Dane county and added more explanation for this observation. Dane county contains 36 Zip codes. It is the second-most populous county in Wisconsin, where the state capital, Madison, is located. Since PHINEX does not cover data from Milwaukee area (Milwaukee county), Dane county is the largest county with collected data in PHINEX. Hence, it indeed distinguishes itself from the remaining area. Zip code similarity values from Dane county tend to be larger than non-Dane zip codes (mean similarities of 15.9 vs 11.2), and the prevalence difference magnitudes are generally smaller for Dane county as well (mean difference magnitudes of -0.710%, range of -6.96% to +6.38%, and SD of 2.66%. See lines 216-225, and Fig 2.

*Information on zip codes and differences in obesity prevalence could be provided in a table or groups of zip-codes with similar characteristics could be grouped together. Range of differences across ZIP codes is quite large, the figure 1 implies less variability in the PHINEX data estimates. Any methodological explanation? Missingness and little data seems to be higher in areas of high risk populations, please expand on this issue.*

**Response**: Sorry for the confusion. Fig 1 presents the data coverage of PHINEX and WHA data, but not the estimated prevalence. We now present a figure showing the differences in estimated obesity prevalence, and in general, the differences are small, see Fig 3. The PHINEX data, with emphasis in Dane county, cover higher SES area. WHA has better representation in other counties/zips, but is still sparse in certain areas and completely missing for Milwaukee. It would be great in the future if we can integrate data from other systems which cover the areas with lower SES. We have expanded the discussion in the discussion section. See lines 277-279.

*The authors conclude, that for similar data the two methods are quite comparable in their estimates, but not where they differ. What is the expected public health impact of one or the other method, considering the bias, provided it were to be used for surveillance?*

**Response**: We have provided an additional figure showing the differences in prevalence, see Fig 3. We suggest that results from multiple methods should be carefully considered. If there are discrepancies between these methods, we should refer to other resources and expert opinions in making decisions. We have added this to the discussion section. See lines 279-282.

*Figures:
The figure 2 in the paper on page 24 (similarities index and difference magnitude magnitude) does not correspond to the description of figure 2 (differences in estimates of obesity using PHINEX and WHA data) on page 11; on page 12 another figure 2 is introduced, corresponding to page 24.*

**Response**: Thank you for catching this. We have fixed it. Now Fig 2 shows prevalence difference estimates versus zip code similarity indices between WHA and PHINEX, and Fig 3 corresponds to the description of the magnitude of prevalence difference estimates (between the process in this paper and WHA) across the State of Wisconsin.

*Minor
Discussion
Pg 13, line 246 add references for health ranking*

**Response**: The reference has been added (reference 31, County Health Rankings for Dane County, Wisconsin. County Health Rankings & Roadmaps. http://www.countyhealthrankings.org/app. Accessed May 10, 2019.). See line 269.

*Pg 13, line 249 “thus it has higher SES…” confusing for reader, replace it with PHINEX*

**Response**: This has been fixed, see line 271.

*Figure 1 should not follow figure 2 in the order of figures in the manuscript. Also, it is placed in the discussion which the reviewer finds odd.*

**Response**: This has been fixed.

*When submitting your revision, we need you to address these additional requirements.*

*1. Please ensure that your manuscript meets PLOS ONE's style requirements, including those for file naming. The PLOS ONE style templates can be found at* [*https://journals.plos.org/plosone/s/file?id=wjVg/PLOSOne_formatting_sample_main_body.pdf*](https://urldefense.proofpoint.com/v2/url?u=https-3A__journals.plos.org_plosone_s_file-3Fid-3DwjVg_PLOSOne-5Fformatting-5Fsample-5Fmain-5Fbody.pdf&d=DwQGaQ&c=eRAMFD45gAfqt84VtBcfhQ&r=aCmD-MMAgPP5KyQFdCwHdbO1Ku4VEKGYihWbBXoltMA&m=nGWLRrlalBjI_SgsDgxSbdR4dhQuWdxBPvfc4y4IaFk&s=zF8xdhYzaAyinWLDmFkGHSt5_q_fifvq2VK-fDK89cM&e=) *and* [*https://journals.plos.org/plosone/s/file?id=ba62/PLOSOne_formatting_sample_title_authors_affiliations.pdf*](https://urldefense.proofpoint.com/v2/url?u=https-3A__journals.plos.org_plosone_s_file-3Fid-3Dba62_PLOSOne-5Fformatting-5Fsample-5Ftitle-5Fauthors-5Faffiliations.pdf&d=DwQGaQ&c=eRAMFD45gAfqt84VtBcfhQ&r=aCmD-MMAgPP5KyQFdCwHdbO1Ku4VEKGYihWbBXoltMA&m=nGWLRrlalBjI_SgsDgxSbdR4dhQuWdxBPvfc4y4IaFk&s=J432GHg4Km02GI_xvoINBdDM4ZGfFAcuDKjGP1FG_Jk&e=)

**Response:** We have followed PLOS ONE’s style requirements.

*2. We note that you have indicated that data from this study are available upon request. PLOS only allows data to be available upon request if there are legal or ethical restrictions on sharing data publicly. For more information on unacceptable data access restrictions, please see* [*http://journals.plos.org/plosone/s/data-availability#loc-unacceptable-data-access-restrictions*](https://urldefense.proofpoint.com/v2/url?u=http-3A__journals.plos.org_plosone_s_data-2Davailability-23loc-2Dunacceptable-2Ddata-2Daccess-2Drestrictions&d=DwQGaQ&c=eRAMFD45gAfqt84VtBcfhQ&r=aCmD-MMAgPP5KyQFdCwHdbO1Ku4VEKGYihWbBXoltMA&m=nGWLRrlalBjI_SgsDgxSbdR4dhQuWdxBPvfc4y4IaFk&s=AlP4sfsqt7M6gQIVC9682w9QNZN1_CAYqbtv-hP5Kvc&e=)*.*

*In your revised cover letter, please address the following prompts:*

*a) If there are ethical or legal restrictions on sharing a de-identified data set, please explain them in detail (e.g., data contain potentially sensitive information, data are owned by a third-party organization, etc.) and who has imposed them (e.g., an ethics committee). Please also provide contact information for a data access committee, ethics committee, or other institutional body to which data requests may be sent.*

*b) If there are no restrictions, please upload the minimal anonymized data set necessary to replicate your study findings as either Supporting Information files or to a stable, public repository and provide us with the relevant URLs, DOIs, or accession numbers. For a list of acceptable repositories, please see* [*http://journals.plos.org/plosone/s/data-availability#loc-recommended-repositories*](https://urldefense.proofpoint.com/v2/url?u=http-3A__journals.plos.org_plosone_s_data-2Davailability-23loc-2Drecommended-2Drepositories&d=DwQGaQ&c=eRAMFD45gAfqt84VtBcfhQ&r=aCmD-MMAgPP5KyQFdCwHdbO1Ku4VEKGYihWbBXoltMA&m=nGWLRrlalBjI_SgsDgxSbdR4dhQuWdxBPvfc4y4IaFk&s=pb4NpNS4jk5Y8a4P6uIpstQYskcBY2coFTecOS7aXvA&e=)*.*

*We will update your Data Availability statement on your behalf to reflect the information you provide.*

**Response:** Because individual subject geographic information below the state level (e.g. city, county, zip code)is protected health information (PHI), we cannot share the raw PHINEX data used in this research. Interested readers could contact UW IRB committee for details.

*3. Thank you for stating the following in the Acknowledgments Section of your manuscript:*

*'This work was supported by funding from the National Institutes of Health (NIH) (grant nos.
R21 HD086754 and P30 CA01570 ).*

*We note that you have provided funding information that is not currently declared in your Funding Statement. However, funding information should not appear in the Acknowledgments section or other areas of your manuscript. We will only publish funding information present in the Funding Statement section of the online submission form.*

*Please remove any funding-related text from the manuscript and let us know how you would like to update your Funding Statement. Currently, your Funding Statement reads as follows:*

*'The work is supported by R21HD086754.'*

**Response:** The funding statement looks good.

*4. We note that Figure 1 in your submission contain [map/satellite] images which may be copyrighted. All PLOS content is published under the Creative Commons Attribution License (CC BY 4.0), which means that the manuscript, images, and Supporting Information files will be freely available online, and any third party is permitted to access, download, copy, distribute, and use these materials in any way, even commercially, with proper attribution. For these reasons, we cannot publish previously copyrighted maps or satellite images created using proprietary data, such as Google software (Google Maps, Street View, and Earth). For more information, see our copyright guidelines:* [*http://journals.plos.org/plosone/s/licenses-and-copyright*](https://urldefense.proofpoint.com/v2/url?u=http-3A__journals.plos.org_plosone_s_licenses-2Dand-2Dcopyright&d=DwQGaQ&c=eRAMFD45gAfqt84VtBcfhQ&r=aCmD-MMAgPP5KyQFdCwHdbO1Ku4VEKGYihWbBXoltMA&m=nGWLRrlalBjI_SgsDgxSbdR4dhQuWdxBPvfc4y4IaFk&s=FLD_yC7CJg4XpyMz90gdx6ejxIdBxFptvNRLt_HCHlE&e=)*.*

*We require you to either (1) present written permission from the copyright holder to publish these figures specifically under the CC BY 4.0 license, or (2) remove the figures from your submission:*

*1.    You may seek permission from the original copyright holder of Figure 1 to publish the content specifically under the CC BY 4.0 license.*

*We recommend that you contact the original copyright holder with the Content Permission Form (*[*http://journals.plos.org/plosone/s/file?id=7c09/content-permission-form.pdf*](https://urldefense.proofpoint.com/v2/url?u=http-3A__journals.plos.org_plosone_s_file-3Fid-3D7c09_content-2Dpermission-2Dform.pdf&d=DwQGaQ&c=eRAMFD45gAfqt84VtBcfhQ&r=aCmD-MMAgPP5KyQFdCwHdbO1Ku4VEKGYihWbBXoltMA&m=nGWLRrlalBjI_SgsDgxSbdR4dhQuWdxBPvfc4y4IaFk&s=yYna-7PE8DnA9M997oRptNg43LH5-6P9NXr3QKut6VU&e=)*) and the following text:*

*“I request permission for the open-access journal PLOS ONE to publish XXX under the Creative Commons Attribution License (CCAL) CC BY 4.0 (*[*http://creativecommons.org/licenses/by/4.0/*](https://urldefense.proofpoint.com/v2/url?u=http-3A__creativecommons.org_licenses_by_4.0_&d=DwQGaQ&c=eRAMFD45gAfqt84VtBcfhQ&r=aCmD-MMAgPP5KyQFdCwHdbO1Ku4VEKGYihWbBXoltMA&m=nGWLRrlalBjI_SgsDgxSbdR4dhQuWdxBPvfc4y4IaFk&s=DhXCPLOCEcxayaIhnI0BrjH-Vv7dHA3ixxkdRMe0zEk&e=)*). Please be aware that this license allows unrestricted use and distribution, even commercially, by third parties. Please reply and provide explicit written permission to publish XXX under a CC BY license and complete the attached form.”*

*Please upload the completed Content Permission Form or other proof of granted permissions as an "Other" file with your submission.*

*In the figure caption of the copyrighted figure, please include the following text: “Reprinted from [ref] under a CC BY license, with permission from [name of publisher], original copyright [original copyright year].”*

*2.    If you are unable to obtain permission from the original copyright holder to publish these figures under the CC BY 4.0 license or if the copyright holder’s requirements are incompatible with the CC BY 4.0 license, please either i) remove the figure or ii) supply a replacement figure that complies with the CC BY 4.0 license. Please check copyright information on all replacement figures and update the figure caption with source information. If applicable, please specify in the figure caption text when a figure is similar but not identical to the original image and is therefore for illustrative purposes only.*

*The following resources for replacing copyrighted map figures may be helpful:*

*USGS National Map Viewer (public domain):* [*http://viewer.nationalmap.gov/viewer/*](https://urldefense.proofpoint.com/v2/url?u=http-3A__viewer.nationalmap.gov_viewer_&d=DwQGaQ&c=eRAMFD45gAfqt84VtBcfhQ&r=aCmD-MMAgPP5KyQFdCwHdbO1Ku4VEKGYihWbBXoltMA&m=nGWLRrlalBjI_SgsDgxSbdR4dhQuWdxBPvfc4y4IaFk&s=ZEeMLqZ0vhIB56iQAgzivO92vHiQJSuPq6MfVTA6adg&e=)

*The Gateway to Astronaut Photography of Earth (public domain):* [*http://eol.jsc.nasa.gov/sseop/clickmap/*](https://urldefense.proofpoint.com/v2/url?u=http-3A__eol.jsc.nasa.gov_sseop_clickmap_&d=DwQGaQ&c=eRAMFD45gAfqt84VtBcfhQ&r=aCmD-MMAgPP5KyQFdCwHdbO1Ku4VEKGYihWbBXoltMA&m=nGWLRrlalBjI_SgsDgxSbdR4dhQuWdxBPvfc4y4IaFk&s=xdfyXbrDHBVCjrrRbHFFpLUKX3OsUDmMDO5TUA70HOA&e=)

*Maps at the CIA (public domain):* [*https://www.cia.gov/library/publications/the-world-factbook/index.html*](https://urldefense.proofpoint.com/v2/url?u=https-3A__www.cia.gov_library_publications_the-2Dworld-2Dfactbook_index.html&d=DwQGaQ&c=eRAMFD45gAfqt84VtBcfhQ&r=aCmD-MMAgPP5KyQFdCwHdbO1Ku4VEKGYihWbBXoltMA&m=nGWLRrlalBjI_SgsDgxSbdR4dhQuWdxBPvfc4y4IaFk&s=45h6GUgrV-YqkSeAVW78r4uIdzQeHUK7EHQ352sJPn0&e=) *and* [*https://www.cia.gov/library/publications/cia-maps-publications/index.html*](https://urldefense.proofpoint.com/v2/url?u=https-3A__www.cia.gov_library_publications_cia-2Dmaps-2Dpublications_index.html&d=DwQGaQ&c=eRAMFD45gAfqt84VtBcfhQ&r=aCmD-MMAgPP5KyQFdCwHdbO1Ku4VEKGYihWbBXoltMA&m=nGWLRrlalBjI_SgsDgxSbdR4dhQuWdxBPvfc4y4IaFk&s=aqQSpx-dEH0m7CoE8TwacdHrZyf7zPxN0CwFpgEMOpM&e=)

*NASA Earth Observatory (public domain):* [*http://earthobservatory.nasa.gov/*](https://urldefense.proofpoint.com/v2/url?u=http-3A__earthobservatory.nasa.gov_&d=DwQGaQ&c=eRAMFD45gAfqt84VtBcfhQ&r=aCmD-MMAgPP5KyQFdCwHdbO1Ku4VEKGYihWbBXoltMA&m=nGWLRrlalBjI_SgsDgxSbdR4dhQuWdxBPvfc4y4IaFk&s=m6VbC13ZiI0v30ux2qqZ1a_CkKxdiO3fWQN68wSFEBc&e=)

*Landsat:* [*http://landsat.visibleearth.nasa.gov/*](https://urldefense.proofpoint.com/v2/url?u=http-3A__landsat.visibleearth.nasa.gov_&d=DwQGaQ&c=eRAMFD45gAfqt84VtBcfhQ&r=aCmD-MMAgPP5KyQFdCwHdbO1Ku4VEKGYihWbBXoltMA&m=nGWLRrlalBjI_SgsDgxSbdR4dhQuWdxBPvfc4y4IaFk&s=zhnlC2XM2KtvNHH99HayYd9GUJAPQUmiUkDbjX2eTuY&e=)

*USGS EROS (Earth Resources Observatory and Science (EROS) Center) (public domain):* [*http://eros.usgs.gov/#*](https://urldefense.proofpoint.com/v2/url?u=http-3A__eros.usgs.gov_-23&d=DwQGaQ&c=eRAMFD45gAfqt84VtBcfhQ&r=aCmD-MMAgPP5KyQFdCwHdbO1Ku4VEKGYihWbBXoltMA&m=nGWLRrlalBjI_SgsDgxSbdR4dhQuWdxBPvfc4y4IaFk&s=ec1kZApHEZmNpWHaBx2fpx3cFnp6cF_svvIvIVlJIZ8&e=)

*Natural Earth (public domain):* [*http://www.naturalearthdata.com/*](https://urldefense.proofpoint.com/v2/url?u=http-3A__www.naturalearthdata.com_&d=DwQGaQ&c=eRAMFD45gAfqt84VtBcfhQ&r=aCmD-MMAgPP5KyQFdCwHdbO1Ku4VEKGYihWbBXoltMA&m=nGWLRrlalBjI_SgsDgxSbdR4dhQuWdxBPvfc4y4IaFk&s=RjQgWpOKHKaskvyRJHlcmys2M7pZHpDU5ma8ZfnZv0c&e=)

**Response:** The figures in our paper are generated using R. We have added references for the R packages we used (see lines 160-163 and references 22-27).
